# Efficacy of Probiotic Supplementation for Preventing Pediatric Urinary Tract Infections: A GRADE‐Based Dose–Response Meta‐Analysis

**DOI:** 10.1002/hsr2.73107

**Published:** 2026-09-01

**Authors:** Sayed Yousef Mojtahedi, Masoumeh Ghasempour, Maryam Ghodsi, Paniz Pourpashang

**Affiliations:** ^1^ Pediatric Chronic Kidney Disease Research Center, Gene, Cell and Tissue Research Institute Tehran University of Medical Sciences Tehran Iran; ^2^ Department of Pediatric Nephrology, Bahrami Hospital, School of Medicine Tehran University Medical Sciences Tehran Iran; ^3^ Department of Pediatric Pulmonology Tehran University of Medical Sciences, Bahrami Hospital, School of Medicine Tehran Iran; ^4^ Department of Pediatrics, School of Medicine, Bahrami Hospital Tehran University Medical Sciences Tehran Iran

**Keywords:** child, meta‐analysis, probiotics, recurrence, urinary tract infections

## Abstract

**Background and Aims:**

Probiotic supplementation has been proposed as a non‐antibiotic strategy for preventing urinary tract infections (UTIs) in children, but its efficacy remains uncertain. We evaluated the effects of probiotic supplementation on pediatric UTI incidence or recurrence, explored potential dose–response relationships, and assessed the certainty of the evidence using GRADE.

**Methods:**

This systematic review and meta‐analysis followed PRISMA 2020. Electronic databases were searched from January 1, 1990, through June 5, 2025, without language restrictions. Thirteen randomized controlled trials involving 3129 participants were included. Risk ratios (RRs) with 95% confidence intervals (CIs) were pooled using a random‐effects model. Subgroup and dose–response analyses were exploratory, and certainty of evidence was evaluated using GRADE.

**Results:**

Probiotic supplementation was associated with a modest reduction in the overall risk of UTI incidence or recurrence (RR = 0.93, 95% CI: 0.88–0.99; *p* = 0.02), with substantial heterogeneity (*I* ^2^ = 71.1%). For UTI incidence, the pooled estimate was RR = 0.95 (95% CI: 0.89–1.01; *p* = 0.12); for UTI recurrence, it was RR = 0.89 (95% CI: 0.81–0.99; *p* = 0.03). No statistically significant nonlinear dose–response association was identified (P for nonlinearity = 0.15). Funnel‐plot asymmetry was visually apparent, but Egger's regression test (*p* = 0.21) and Begg's rank‐correlation test (*p* = 0.45) did not provide statistically significant evidence of small‐study effects. Certainty of evidence was low because of risk‐of‐bias and inconsistency concerns.

**Conclusion:**

Probiotic supplementation may be associated with a small reduction in pediatric UTI risk, but substantial clinical and statistical heterogeneity and low‐certainty evidence limit confidence in the pooled estimate. The apparent association with lower UTI recurrence should be interpreted cautiously, and the evidence does not establish an optimal probiotic dose or equivalent efficacy across strains and formulations.

## 1 Introduction

Urinary tract infections (UTIs) are among the most common bacterial infections in children and represent an important cause of morbidity, healthcare utilization, and antibiotic exposure [1, 2]. Pediatric UTIs range from uncomplicated cystitis to febrile pyelonephritis and, particularly when recurrent or associated with underlying urinary tract abnormalities, may contribute to renal scarring and other long‐term complications [3]. The increasing burden of antimicrobial resistance and concerns regarding repeated or prolonged antibiotic exposure have intensified interest in safe, non‐antibiotic approaches for UTI prevention. Within this context, probiotics have emerged as a potential microbiota‐directed strategy because of their capacity to influence microbial community structure and host–microbe interactions [4, 5].

The biological rationale for probiotic supplementation extends beyond simple competitive exclusion of pathogens. Probiotic effects are increasingly understood to be strain specific and may involve modulation of the gut and mucosal microbiota, production of bioactive microbial metabolites, enhancement of epithelial barrier integrity, and regulation of local and systemic immune responses [6]. Recent mechanistic evidence summarized by Adhikary et al. indicates that probiotic‐ and synbiotic‐mediated effects may involve microbial metabolites capable of influencing inflammatory signaling, redox homeostasis, epithelial barrier function, and host immune responses [7]. These mechanisms provide biological plausibility for a potential role of probiotics in host defense against infection; however, they should not be interpreted as direct evidence of efficacy in preventing pediatric UTIs. In the urinary tract setting, additional proposed mechanisms include interference with uropathogen adhesion, competition for ecological niches, and production of antimicrobial compounds such as organic acids. Nevertheless, whether these mechanistic properties translate into clinically meaningful prevention of pediatric UTI remains uncertain [5, 8].

Clinical evidence, however, has remained inconsistent. Previous systematic reviews and randomized trials have reported heterogeneous findings, potentially reflecting differences in probiotic strains, formulations, administered doses, treatment duration, underlying UTI risk, concomitant antibiotic prophylaxis, and outcome definitions [5, 9]. These uncertainties are particularly relevant because probiotic effects cannot necessarily be generalized across strains or inferred solely from total CFU exposure. Accordingly, a quantitative synthesis that considers both overall efficacy and potential dose–response patterns, while formally evaluating the certainty of evidence, is warranted [10, 11].

Accordingly, this systematic review and meta‐analysis evaluated the efficacy of probiotic supplementation for preventing UTI incidence or recurrence in children, with particular attention to potential dose–response relationships. We additionally applied the GRADE framework to assess the certainty of the available evidence. By synthesizing randomized controlled trials, this review aimed to clarify the potential role of probiotics in pediatric UTI prevention and to identify sources of clinical and methodological variability relevant to interpretation and future trial design.

## 2 Methods

### 2.1 Protocol and Registration

The review methods followed the Cochrane Handbook for Systematic Reviews of Interventions [12], and reporting followed the Preferred Reporting Items for Systematic Reviews and Meta‐Analyses (PRISMA) 2020 statement [13]. The review was not prospectively registered in PROSPERO, and no separate peer‐reviewed protocol was published.

### 2.2 Information Sources and Search Strategy

A comprehensive literature search was conducted to identify randomized controlled trials evaluating probiotic supplementation for the prevention of urinary tract infection (UTI) incidence or recurrence in pediatric populations. PubMed/MEDLINE, Embase, the Cochrane Central Register of Controlled Trials (CENTRAL), Scopus, and Web of Science were searched for studies published from January 1, 1990, through June 5, 2025. No language restrictions were applied. ClinicalTrials.gov and the World Health Organization International Clinical Trials Registry Platform (WHO ICTRP) were additionally searched to identify ongoing, completed, or unpublished trials, and gray‐literature sources included Google Scholar and ProQuest Dissertations & Theses Global.

The search strategy was developed using database‐specific controlled vocabulary, including Medical Subject Headings (MeSH) and equivalent indexing terms, together with relevant free‐text terms. Search concepts encompassed urinary tract infection and related conditions (e.g., UTI, cystitis, and pyelonephritis), probiotic interventions and commonly studied probiotic genera (e.g., *Lactobacillus*, *Bifidobacterium*, and *Saccharomyces*), and pediatric populations (e.g., child, infant, newborn, adolescent, pediatric, and pediatric). Synbiotic‐ and symbiotic‐related terms were intentionally included in the intervention search block to maximize retrieval sensitivity because potentially relevant records may be indexed using broader microbiome‐intervention terminology or may include a separable probiotic‐only treatment arm. Their inclusion in the search strategy did not broaden the eligibility criteria: studies evaluating synbiotics as the sole intervention were excluded unless a probiotic‐only intervention arm or an effect attributable specifically to the probiotic component could be extracted separately. Boolean operators, truncation, and database‐specific syntax were used as appropriate.

To identify additional eligible studies, the reference lists of included trials and relevant systematic reviews were manually screened, and forward citation tracking was performed using Web of Science and Scopus. When potentially eligible studies contained insufficient or unclear information, corresponding authors were contacted for clarification or additional data when necessary. The complete database‐specific search strategies, including controlled vocabulary, free‐text terms, Boolean operators, and applied limits, are provided in Supporting Information S1: Table 1 to facilitate reproducibility.

### 2.3 Ethics Approval and Informed Consent

This systematic review and meta‐analysis used published aggregate data and involved no new recruitment, intervention, collection of identifiable participant‐level data, or contact with study participants. Accordingly, institutional ethics approval and individual informed consent were not required for this review.

### 2.4 Eligibility Criteria

Eligibility was defined a priori according to the Population, Intervention, Comparator, Outcome, and Study design (PICOS) framework. Eligible studies included randomized controlled trials enrolling pediatric participants aged 0–18 years who were at risk of developing a urinary tract infection (UTI) or had a history of UTI, including children with recurrent UTI, vesicoureteral reflux, acute pyelonephritis, or other pediatric clinical conditions in which UTI prevention was evaluated. Studies including mixed adult and pediatric populations were eligible only when pediatric data could be extracted separately. Animal studies and studies conducted exclusively in adults were excluded.

Eligible interventions consisted of viable probiotic microorganisms administered alone or in addition to standard care, provided that the probiotic component could be clearly identified. Trials evaluating single‐strain or multi‐strain probiotic preparations were eligible irrespective of the specific microorganism or formulation. Studies evaluating synbiotics, prebiotics, postbiotics, or non‐viable microbial products as the sole intervention were excluded. Multi‐component or multi‐arm studies were considered eligible only when a probiotic‐only intervention arm, or an effect specifically attributable to the probiotic intervention, could be extracted separately. This distinction was maintained despite the inclusion of synbiotic‐ and symbiotic‐related terms in the search strategy, which were used solely to maximize retrieval sensitivity.

Eligible comparator groups included placebo, no probiotic intervention, or standard care, including antibiotic prophylaxis when this represented usual clinical management. Trials were included only when the comparison permitted estimation of the effect attributable to probiotic supplementation. Studies without a concurrent comparison group or those in which the probiotic effect could not be isolated from other co‐interventions were excluded.

The primary outcome was the incidence or recurrence of UTI during follow‐up, as defined by the original investigators using clinical and/or microbiological criteria. Studies were eligible for quantitative synthesis when sufficient dichotomous outcome data were available, or could be derived, to calculate a risk ratio and corresponding variance. Trials meeting the review eligibility criteria but lacking sufficient information for meta‐analysis were retained for qualitative consideration when appropriate rather than being excluded solely because quantitative pooling was not possible.

Only randomized controlled trials were eligible. Non‐randomized intervention studies, observational studies, case–control studies, cohort studies, cross‐sectional studies, case reports, case series, reviews, editorials, and preclinical studies were excluded. Conference abstracts were considered only when sufficient methodological and outcome data were available to establish eligibility and derive the required effect estimate, either from the abstract itself or through supporting information or author contact. Duplicate or overlapping publications were reconciled so that each trial contributed only once to a given analysis, with the most complete report used as the primary source of data.

No language or geographic restrictions were applied. Eligibility decisions were made independently by two reviewers during title/abstract and full‐text screening. Disagreements were resolved through discussion and, when necessary, adjudication by a third reviewer. Studies were not excluded on the basis of methodological quality alone; risk of bias was assessed separately after study inclusion using the Cochrane Risk of Bias Tool.

### 2.5 Study Selection

A multi‐stage approach was employed to identify and select studies evaluating the efficacy of probiotic supplementation for preventing UTIs in pediatric populations.

All records retrieved from the electronic database searches, gray literature sources, and additional hand‐searching were imported into a reference management software (EndNote X9) to facilitate organization and remove duplicates. Following deduplication, the remaining records were uploaded to a systematic review management tool (Covidence) to streamline the screening process. Two independent reviewers (P.P. and S.Y.M.) screened titles and abstracts against the predefined eligibility criteria outlined in the PICOS framework. Studies that appeared to meet the inclusion criteria or required further evaluation were advanced to full‐text review.

During full‐text screening, the same two reviewers (P.P. and S.Y.M.) independently assessed each report for eligibility and documented reasons for exclusion. Disagreements were resolved through discussion and, when consensus could not be reached, adjudicated by a third reviewer (M.G.). Study authors were contacted when additional clarification was necessary to determine eligibility or extract relevant data.

**Figure 1:**
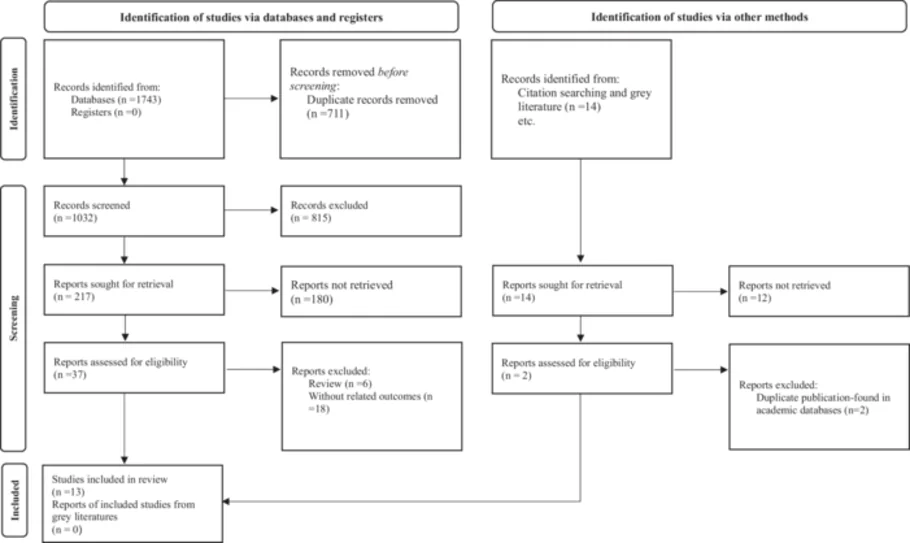
PRISMA 2020 flow diagram of study identification, screening, eligibility assessment, and inclusion.

The study‐selection process was documented using a PRISMA 2020 flow diagram, including the numbers of records identified, screened, assessed for eligibility, excluded with reasons, and included in the final synthesis (Figure 1).

### 2.6 Data Extraction

A standardized data extraction form was developed a priori and piloted on a subset of three studies to ensure clarity and consistency before full implementation. The form was tailored to capture data aligned with the objectives of evaluating the efficacy of probiotic supplementation for preventing UTIs in pediatric populations, with a focus on dose–response relationships.

Data extraction was performed independently by two reviewers (M.Gh. and P.P.) using a standardized data‐extraction form. Disagreements were resolved through discussion and, when necessary, consultation with a third reviewer (M.G.). Extracted information included study characteristics, participant characteristics, details of the probiotic intervention and comparator, outcome definitions and event data, follow‐up duration, and methodological characteristics relevant to the risk‐of‐bias assessment. The extracted data were compiled in Microsoft Excel and checked for consistency before statistical analysis.

### 2.7 Risk of Bias Assessment

Risk of bias in the included randomized controlled trials was independently assessed by two reviewers (S.Y.M. and M.Gh.) using the Cochrane Risk of Bias Tool, version 1 (RoB 1), in accordance with guidance from the Cochrane Handbook for Systematic Reviews of Interventions [12]. Seven domains were evaluated: random sequence generation, allocation concealment, blinding of participants and personnel, blinding of outcome assessment, incomplete outcome data, selective reporting, and other potential sources of bias. Each domain was classified as low, high, or unclear risk of bias based on the information reported in the trial publications and associated materials. Disagreements between reviewers were resolved through discussion and, when consensus could not be reached, by adjudication with a third reviewer (M.G.). When reporting was insufficient to support a definitive judgment, supporting materials and available trial‐registration records were examined, and study authors were contacted for clarification when appropriate. Risk‐of‐bias judgments were made at the domain level rather than by assigning an overall numerical quality score, and the resulting assessments are summarized in Table 2.

### 2.8 Statistical Analysis

For the primary outcome, defined as the incidence or recurrence of urinary tract infection (UTI), treatment effects were expressed as risk ratios (RRs) with 95% confidence intervals (CIs). Although the included trials differed in probiotic strain composition, single‐ versus multi‐strain formulations, administered doses, dosing schedules, supplementation duration, clinical setting, and concomitant therapies, they addressed a common clinical question: whether probiotic supplementation reduces the risk of UTI incidence or recurrence in pediatric populations compared with placebo, no probiotic supplementation, or standard care. Accordingly, quantitative synthesis was undertaken to estimate an average class‐level effect of probiotic supplementation, rather than a strain‐ or product‐specific treatment effect. This approach did not assume biological or therapeutic equivalence among the individual probiotic preparations.

Study‐specific RRs were pooled using a random‐effects model with the DerSimonian–Laird estimator because genuine between‐study variation in treatment effects was anticipated owing to differences in probiotic composition and formulation, administered dose, supplementation duration, participant characteristics, underlying UTI risk, concomitant antimicrobial therapy, clinical setting, and follow‐up. Statistical heterogeneity was evaluated using Cochran's Q test and quantified using the *I* ^2^ statistic. A *p* value < 0.10 for Cochran's Q was considered indicative of statistically detectable heterogeneity because of the limited power of this test, whereas *I* ^2^ was interpreted in conjunction with the magnitude, direction, and clinical characteristics of the study‐specific effects rather than according to rigid thresholds alone. Given the marked clinical heterogeneity of the included pediatric populations, exploratory subgroup analyses were conducted, when sufficient data were available, according to UTI outcome type (UTI incidence vs. UTI recurrence) and supplementation duration (< 6 months vs. ≥ 6 months). More narrowly defined clinical populations, including children with vesicoureteral reflux, acute pyelonephritis, critically ill children admitted to pediatric intensive care units, and preterm infants, were not analyzed as separate subgroups when the number of available studies was insufficient to support reliable pooled estimates. Similarly, because of the diversity of probiotic preparations and the limited number of studies evaluating individual strains or formulations, strain‐specific pooled estimates were not interpreted as demonstrating comparative efficacy between probiotic species or products. All subgroup findings were therefore considered exploratory rather than confirmatory.

An exploratory dose–response analysis was performed to investigate whether the magnitude of the association between probiotic supplementation and UTI incidence or recurrence varied according to the administered probiotic dose. Because the included interventions differed substantially in strain composition, formulation, and dosing frequency, probiotic exposure was harmonized as the total daily viable microbial dose expressed in colony‐forming units per day (CFU/day). When the reported CFU referred to a single administration, the dose was multiplied by the reported number of administrations per day. For multi‐strain formulations, strain‐specific viable counts were summed when the CFU contribution of each strain was available; when only the aggregate viable count of the formulation was reported, the reported total CFU was used. For preparations reported as CFU per unit volume or mass, the administered volume or mass and dosing frequency were used to derive the corresponding total daily exposure whenever sufficient information was available. Studies for which a fixed total daily CFU exposure could not be reliably derived without unsupported assumptions, including incompletely specified or exclusively weight‐based regimens that could not be converted to a study‐level daily dose, were not included in the dose–response model.

Because probiotic doses spanned several orders of magnitude, the harmonized daily exposure was expressed on a log10 CFU/day scale for dose–response modeling. Study‐specific log‐transformed risk ratios [ln(RR)] were modeled as a function of probiotic dose, and restricted cubic splines were used to assess potential non‐linearity in the dose–response relationship following established dose–response meta‐analytic methods [14]. Evidence of non‐linearity was evaluated by testing the spline terms beyond the linear component. This analysis was considered exploratory and hypothesis‐generating, because CFU harmonization provides a common quantitative measure of viable microbial exposure but does not establish biological equivalence or comparable potency across different probiotic strains, combinations, or formulations. Consequently, the dose–response findings were not interpreted as defining an optimal probiotic dose applicable across products.

The robustness of the overall pooled estimate was examined using leave‐one‐out sensitivity analysis, whereby each study was sequentially omitted and the meta‐analysis repeated to determine whether any individual trial disproportionately influenced the pooled RR or between‐study heterogeneity. Quantitative analyses were based on outcome data available in the published reports and associated supporting materials. When information required for synthesis was unclear or incompletely reported, trial registrations and supporting sources were reviewed and study authors were contacted for clarification when necessary. Unsupported assumptions regarding unavailable outcome data were avoided whenever possible, and any assumptions required for analysis were documented.

Potential small‐study effects were assessed for the primary meta‐analysis because at least 10 studies were available. Funnel‐plot symmetry was evaluated visually and supplemented by Egger's regression test and Begg's rank‐correlation test. These analyses were interpreted cautiously, recognizing that funnel‐plot asymmetry may arise from sources other than publication bias, including clinical heterogeneity, methodological differences, and variation in study precision.

The overall pooled analysis was the primary analysis; subgroup and dose–response analyses were exploratory. Cochran's Q test was used to assess between‐study heterogeneity, and a Cochran Q test for subgroup differences was used for subgroup comparisons. Potential dose–response nonlinearity was evaluated by testing the spline terms beyond the linear component. Egger's regression test and Begg's rank‐correlation test were used to assess small‐study effects. All statistical tests were two‐sided. A two‐sided *p* < 0.05 was considered statistically significant for primary and exploratory inferential tests unless otherwise specified; *p* < 0.10 was used for Cochran's Q because of its limited power. Analyses were performed in R version 4.4.0 (R Foundation for Statistical Computing, Vienna, Austria), using the meta package for conventional meta‐analysis and sensitivity analyses and the dosresmeta package for dose–response modeling. Statistical reporting was reviewed for consistency with the recommendations of Assel et al. [15] and the SAMPL guidelines [16]. The certainty of evidence for the primary outcome was evaluated using the GRADE framework across risk of bias, inconsistency, indirectness, imprecision, and publication bias, with judgments summarized in a GRADE evidence profile.

## 3 Results

### 3.1 Study Characteristics

After removal of duplicates from 1743 records, 1032 unique records remained for screening, and 13 eligible studies from nine countries across three continents were included in the systematic review [17, 18, 19, 20, 21, 22, 23, 24, 25, 26, 27, 28, 29] (Figure 1). The studies were conducted in Iran (*n* = 2), South Korea (*n* = 3), India (*n* = 2), Poland, Slovenia, Turkey, Colombia, the United States, and Italy. They were published between 2002 and 2024 and included 3129 participants in total. Study sample sizes ranged from 30 to 750 participants, and reported follow‐up ranged from very short inpatient periods to 2 years. Key study characteristics are summarized in Table 1.

**Table 1:** Characteristics of the included randomized controlled trials.

Study	Country	Population/clinical setting	Age	Sample size (I/C)	Probiotic strain(s)/formulation and administered dose	Harmonized daily dose (CFU/day)	Comparator	Diagnostic definition of UTI	Follow‐up	Outcome
Daniel 2024	Poland	Children after first UTI	8.5 (5.8–11.0) y	54 (27/27)	*Lactobacillus rhamnosus* PL1 + *Lactobacillus plantarum* PM1; each 1 × 10^9^ CFU once daily	2 × 10^9^/day	Antibiotics	Two consecutive urine cultures positive for a single microorganism (≥ 10^5^ CFU/mL)	6 mo	UTI
Meštrović Popovič 2022	Slovenia	Children with UTI	5.2 (1.4) y	30 (14/16)	*Lactobacillus plantarum* PCS26; 1 × 10^9^ CFU per reported dose	Not reliably derivable^a^	Placebo/control	UTI diagnosis according to the original trial protocol	6 mo	Recurrent UTI
Sadeghi‐Bojd 2019	Iran	Children after first febrile UTI	4 mo–5 y	181 (90/91)	Multi‐strain *Lactobacillus*/*Bifidobacterium* preparation; reported regimen includes 10^9^ CFU/10 mL and 20 mL daily	Not reliably derivable^a^	Placebo	Urine‐culture‐based assessment as defined in the original trial	18 mo	UTI
Cetin 2014	Turkey	Children after first UTI	NR	80 (40/40)	*Saccharomyces boulardii* + antibiotics; 5 × 10^9^ CFU twice daily	1 × 10^10^/day	Antibiotic	Positive urine culture for a single microorganism (≥ 10^5^ CFU/mL)	12 mo	UTI; recurrent UTI
Kumar 2013a	India	PICU	3–144 mo	150 (75/75)	Multi‐strain probiotic with fructo‐oligosaccharides; 1.25 × 10^9^ CFU per reported dose	Not reliably derivable^a^	Lactose placebo	Two consecutive urine cultures positive for a single microorganism (≥ 10^5^ CFU/mL)	2 wk	UTI
Kumar 2013b	India	PICU	3–144 mo	720 (344/376)	Multi‐strain probiotic with fructo‐oligosaccharides; 1.25 × 10^9^ CFU per reported dose	Not reliably derivable^a^	Standard care/comparator	Two consecutive urine cultures positive for a single microorganism (≥ 10^5^ CFU/mL)	1 wk	UTI
Mohseni 2013	Iran	Persistent vesicoureteral reflux	36–180 mo	85 (44/41)	*Lactobacillus acidophilus* + *Bifidobacterium lactis*; 10^7^ CFU/mL, 0.25 mL/kg three times daily + nitrofurantoin 1 mg/kg nightly	7.5 × 10^6^ CFU/kg/day^b^	Nitrofurantoin 1 mg/kg nightly	Positive urine culture for a single microorganism (≥ 10^5^ CFU/mL)	2 y	Recurrent UTI
Lee 2015	South Korea	Persistent vesicoureteral reflux	13–36 mo	128 (64/64)	*Lactobacillus acidophilus*; 1 × 10^8^ CFU twice daily	2 × 10^8^/day	Antibiotic	Significant bacteriuria: > 10^3^ CFU/mL in suprapubic aspirate or > 10^5^ CFU/mL in catheterized urine	1 y	Recurrent UTI
Lee 2016	South Korea	Acute pyelonephritis	1–24 mo	186 (113/73)	*Lactobacillus acidophilus*; 1 × 10^8^ CFU twice daily for 6 mo	2 × 10^8^/day	Trimethoprim–sulfamethoxazole/no prophylaxis	Significant bacteriuria in symptomatic infants, as defined in the original study	6 mo	Recurrent UTI
Lee 2007	South Korea	Persistent vesicoureteral reflux	13–36 mo	120 (60/60)	*Lactobacillus acidophilus*; 1 × 10^8^ CFU twice daily for 1 y	2 × 10^8^/day	Trimethoprim–sulfamethoxazole	Significant bacteriuria: > 10^3^ CFU/mL in suprapubic aspirate or > 10^5^ CFU/mL in catheterized urine	12 mo	Recurrent UTI
Rojas 2012	Colombia	PICU	< 48 h	750 (378/372)	*Lactobacillus reuteri* DSM; 1 × 10^8^ CFU once daily for 2 d	1 × 10^8^/day	Oil drops without probiotic	Positive urine culture for a single microorganism (≥ 10^4^ CFU/mL)	2 d	UTI
Honeycutt 2007	USA	PICU	1–216 mo	61 (30/31)	*Lactobacillus rhamnosus* GG; 1 × 10^10^ CFU once daily until discharge	1 × 10^10^/day	Inulin capsule	Two consecutive urine cultures positive for a single microorganism (≥ 10^5^ CFU/mL)	Until discharge	UTI
Dani 2002	Italy	Preterm infants	1 mo	585 (290/295)	Milk + *Lactobacillus GG*; 6 × 10^9^ CFU once daily	6 × 10^9^/day	Milk + placebo	Two consecutive urine cultures positive for a single microorganism (≥ 10^5^ CFU/mL)	1 wk	UTI

*Note*. Data are presented as reported in the current extraction table and harmonized for clarity.

Abbreviations: CFU, colony‐forming units; I/C, intervention/control; mo, months; NR, not reported; PICU, pediatric intensive care unit; UTI, urinary tract infection; wk, weeks; y, years.

^a^ A fixed total CFU/day could not be derived without an unsupported assumption from the information available in the current extraction; these studies should not contribute to the dose–response model unless the administration frequency and formulation are verified from the full‐text report.

^b^ Weight‐based regimen; the exposure is expressed as CFU/kg/day and is not directly comparable with a single fixed CFU/day. For a fixed‐dose dose–response model, this study should be excluded unless a prespecified, defensible conversion based on participant‐level or adequately reported body‐weight data is applied.

### 3.2 Study Population and Intervention Type

The included studies represented diverse pediatric clinical settings, including children with previous or recurrent UTI, vesicoureteral reflux, acute pyelonephritis, critically ill children in pediatric intensive care units, and preterm infants. Probiotic interventions also varied substantially and included single‐ and multi‐strain preparations containing Lactobacillus, Bifidobacterium, Saccharomyces, or combinations of these microorganisms. Comparator regimens ranged from placebo or no probiotic intervention to standard care and antibiotic prophylaxis.

### 3.3 Risk‐of‐Bias Assessment

Risk of bias was assessed across seven domains using the Cochrane Risk of Bias Tool, version 1 (RoB 1), with detailed judgments presented in Table 2. Four trials (Daniel 2024; Kumar 2013a; Kumar 2013b; and Honeycutt 2007) were judged to be at low risk of bias across all assessed domains. Five trials had at least one domain judged to be at high risk of bias: Meštrović Popović (2022) had high‐risk judgments for blinding of participants and personnel and incomplete outcome data; Lee 2015 had a high‐risk judgment for blinding of outcome assessment; Lee 2016 had high‐risk judgments across random sequence generation, allocation concealment, blinding, outcome assessment, incomplete outcome data, and selective reporting; Rojas 2012 had high‐risk judgments for blinding of participants and personnel and other bias; and Dani 2002 had a high‐risk judgment for incomplete outcome data. The remaining studies (Sadeghi‐Bojd 2019; Cetin 2014; Mohseni 2013; and Lee 2007) contained one or more unclear‐risk judgments but no high‐risk domain, largely reflecting insufficient methodological reporting. Overall, these domain‐level concerns contributed to uncertainty in the evidence base and were considered in the subsequent GRADE assessment (Table 3).

**Table 2:** Risk‐of‐bias assessment of the included studies.

Study	Random sequence generation	Allocation concealment	Blinding of participants and personnel	Blinding of outcome assessment	Incomplete outcome data	Selective reporting	Other bias
Daniel 2024	**● Low**	**● Low**	**● Low**	**● Low**	**● Low**	**● Low**	**● Low**
Meštrović Popović 2022	**● Low**	**● Unclear**	**● High**	**● Low**	**● High**	**● Low**	**● Unclear**
Sadeghi‐Bojd 2019	**● Low**	**● Unclear**	**● Unclear**	**● Unclear**	**● Low**	**● Low**	**● Unclear**
Cetin 2014	**● Unclear**	**● Unclear**	**● Unclear**	**● Unclear**	**● Unclear**	**● Unclear**	**● Unclear**
Kumar 2013a	**● Low**	**● Low**	**● Low**	**● Low**	**● Low**	**● Low**	**● Low**
Kumar 2013b	**● Low**	**● Low**	**● Low**	**● Low**	**● Low**	**● Low**	**● Low**
Mohseni 2013	**● Low**	**● Low**	**● Unclear**	**● Unclear**	**● Low**	**● Unclear**	**● Unclear**
Lee 2015	**● Unclear**	**● Unclear**	**● Unclear**	**● High**	**● Unclear**	**● Unclear**	**● Unclear**
Lee 2007	**● Unclear**	**● Unclear**	**● Unclear**	**● Unclear**	**● Unclear**	**● Unclear**	**● Unclear**
Lee 2016	**● High**	**● High**	**● High**	**● High**	**● High**	**● High**	**● Unclear**
Rojas 2012	**● Unclear**	**● Low**	**● High**	**● Unclear**	**● Unclear**	**● Unclear**	**● High**
Honeycutt 2007	**● Low**	**● Low**	**● Low**	**● Low**	**● Low**	**● Low**	**● Low**
Dani 2002	**● Unclear**	**● Unclear**	**● Unclear**	**● Unclear**	**● High**	**● Unclear**	**● Unclear**

Judgment key: ● Low risk ● High risk ● Unclear risk

*Note*. Risk‐of‐bias judgments are presented at the domain level using the Cochrane Risk of Bias Tool, version 1 (RoB 1). No numerical quality score or composite overall score was assigned.

**Table 3:** GRADE evidence profile for the effect of probiotic supplementation on pediatric urinary tract infection incidence or recurrence.

Outcome	No. of studies	Participants	Relative effect (95% CI)	Risk of bias	Inconsistency	Indirectness	Imprecision	Publication bias	Certainty of evidence
UTI incidence or recurrence	13 RCTs	3129	RR 0.93 (0.88–0.99)	Serious^a^	Serious^b^	Not seriousᶜ	Not serious^d^	Not detected^e^	**Low** ⨁⨁ ◯◯

Abbreviations: CI, confidence interval; GRADE, Grading of Recommendations Assessment, Development and Evaluation; RCT, randomized controlled trial; RR, risk ratio; UTI, urinary tract infection.

**^a^Risk of bias:** Downgraded by one level because several included trials had high or unclear risk of bias in important domains, particularly blinding, allocation procedures, incomplete outcome data, and/or selective reporting. These concerns reduced confidence that the pooled estimate was unaffected by methodological limitations.

**^b^Inconsistency:** Downgraded by one level because substantial between‐study heterogeneity was observed in the overall meta‐analysis (*I* ^2^ = 71.1%). The heterogeneity was not adequately explained by the available subgroup analyses and was clinically plausible given differences in pediatric populations, probiotic strains and formulations, comparator regimens, UTI definitions, and follow‐up duration.

**ᶜIndirectness:** Not downgraded because the included trials directly evaluated pediatric populations receiving probiotic supplementation for UTI incidence or recurrence and used clinically relevant comparator groups. Nevertheless, variation across populations and probiotic formulations limits extrapolation to specific strains or clinical settings.

**^d^Imprecision:** Not downgraded because the pooled estimate was based on 3129 participants and the 95% CI was relatively narrow. However, the confidence interval remained close to the null, and the clinical importance of the modest relative effect remains uncertain; no validated minimally important clinical threshold is available for this specific intervention–outcome context.

**^e^Publication bias:** Not downgraded. Although visual inspection of the funnel plot suggested some asymmetry, neither Egger's regression test (*p* = 0.21) nor Begg's rank‐correlation test (*p* = 0.45) provided statistically significant evidence of small‐study effects. These assessments were interpreted cautiously because of the limited number of trials and substantial heterogeneity.

### 3.4 Meta‐Analysis Results

**Figure 2:**
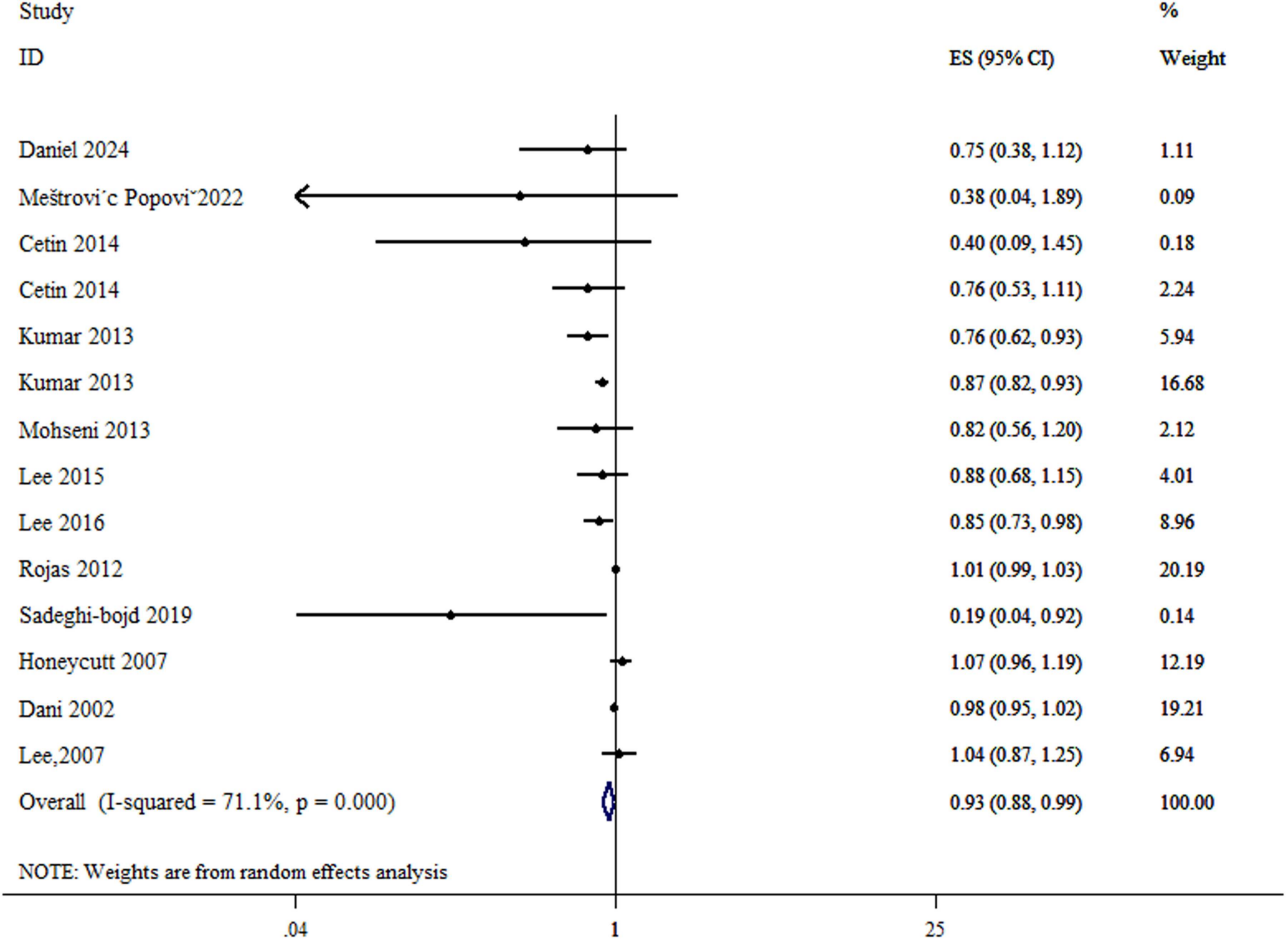
Random‐effects forest plot of the association between probiotic supplementation and pediatric UTI incidence or recurrence. Effect estimates are risk ratios (RRs) with 95% confidence intervals.

The meta‐analysis included 13 studies comprising 3129 participants. Probiotic supplementation was associated with a modest reduction in the risk of UTI incidence or recurrence compared with placebo or standard care (RR = 0.93, 95% CI: 0.88–0.99; *p* = 0.02), with substantial between‐study heterogeneity (*I* ^2^ = 71.1%) (Figure 2). The pooled RR corresponds to an estimated 7% relative risk reduction. Because no validated minimal clinically important difference has been established for probiotic prevention of pediatric UTI, the clinical importance of this relative effect should be interpreted in the context of baseline risk, absolute effects, patient population, and certainty of the evidence rather than an arbitrary percentage threshold.

Substantial between‐study heterogeneity was observed in the overall analysis (*I* ^2^ = 71.1%), indicating variability in treatment effects across studies. Heterogeneity remained substantial among studies evaluating UTI incidence (*I* ^2^ = 80.5%) but was not detected among studies evaluating UTI recurrence (*I* ^2^ = 0.0%). The formal test for subgroup differences did not provide evidence that effects differed by UTI outcome type (*Q* = 1.16, df = 1, *p* = 0.28), and subgrouping by supplementation duration (<6 months vs. ≥6 months) likewise did not materially account for the overall heterogeneity.

### 3.5 Subgroup and Sensitivity Analyses

**Figure 3:**
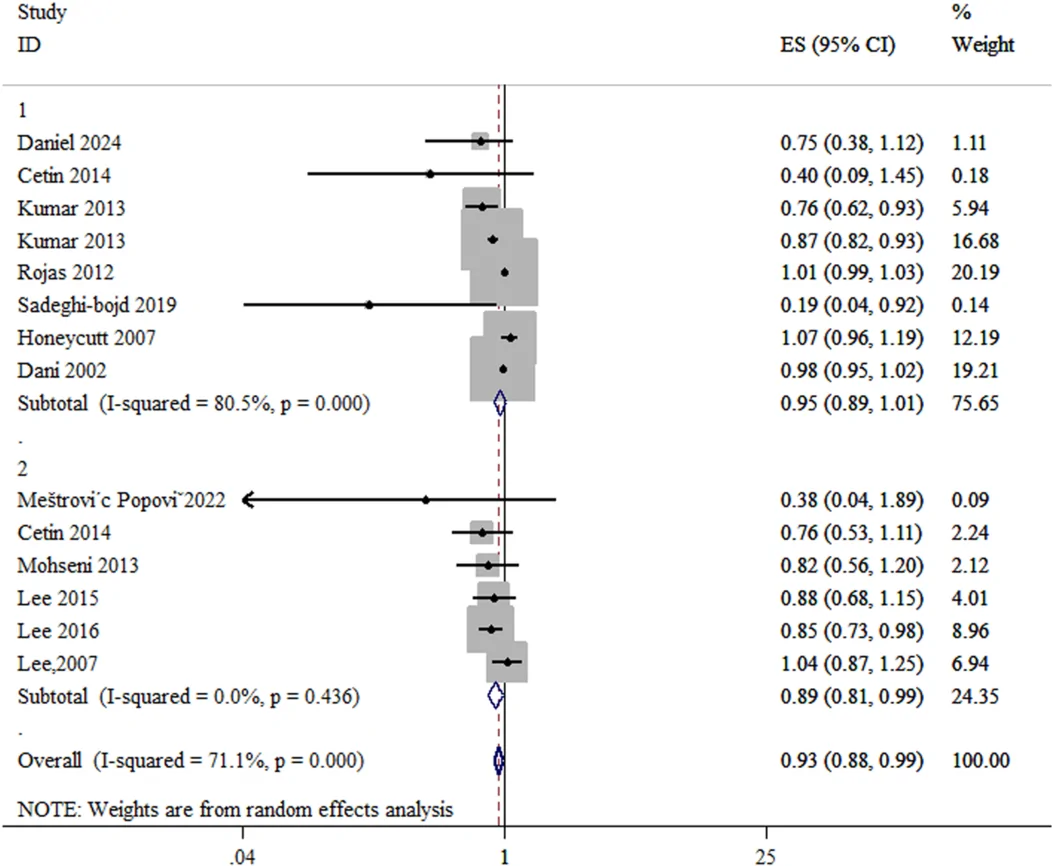
Exploratory subgroup analysis according to UTI outcome type (UTI incidence vs. UTI recurrence). Effect estimates are risk ratios (RRs) with 95% confidence intervals.

Given the clinical diversity of the included studies, exploratory subgroup analyses were conducted according to UTI outcome type. Among studies evaluating UTI incidence, the pooled estimate was RR = 0.95 (95% CI: 0.89–1.01; *p* = 0.12), with substantial within‐subgroup heterogeneity (*I* ^2^ = 80.5%; P for heterogeneity < 0.001). Among studies evaluating UTI recurrence, the pooled estimate was RR = 0.89 (95% CI: 0.81–0.99; *p* = 0.03), with no detectable within‐subgroup heterogeneity (*I* ^2^ = 0.0%; P for heterogeneity = 0.44) (Figure 3). Although the point estimate suggested a somewhat larger relative effect for UTI recurrence, the formal test for subgroup differences was not statistically significant (*Q* = 1.16, df = 1, *p* = 0.28). These findings are exploratory and should be interpreted cautiously given the limited number of studies within each outcome category and residual variability in diagnoses, baseline UTI risk, comparator regimens, concomitant therapies, probiotic formulations, UTI definitions, and follow‐up duration.

An additional exploratory subgroup analysis according to supplementation duration (<6 months vs. ≥6 months) did not identify a statistically significant difference in treatment effects between duration categories and did not materially explain the observed between‐study heterogeneity (Supporting Information S1: Figure 1). Leave‐one‐out sensitivity analysis, in which each study was sequentially omitted from the overall meta‐analysis, showed that no individual study materially altered the direction or magnitude of the pooled effect estimate, supporting the stability of the overall findings (Supporting Information S1: Figure 2).

### 3.6 Publication Bias

**Figure 4:**
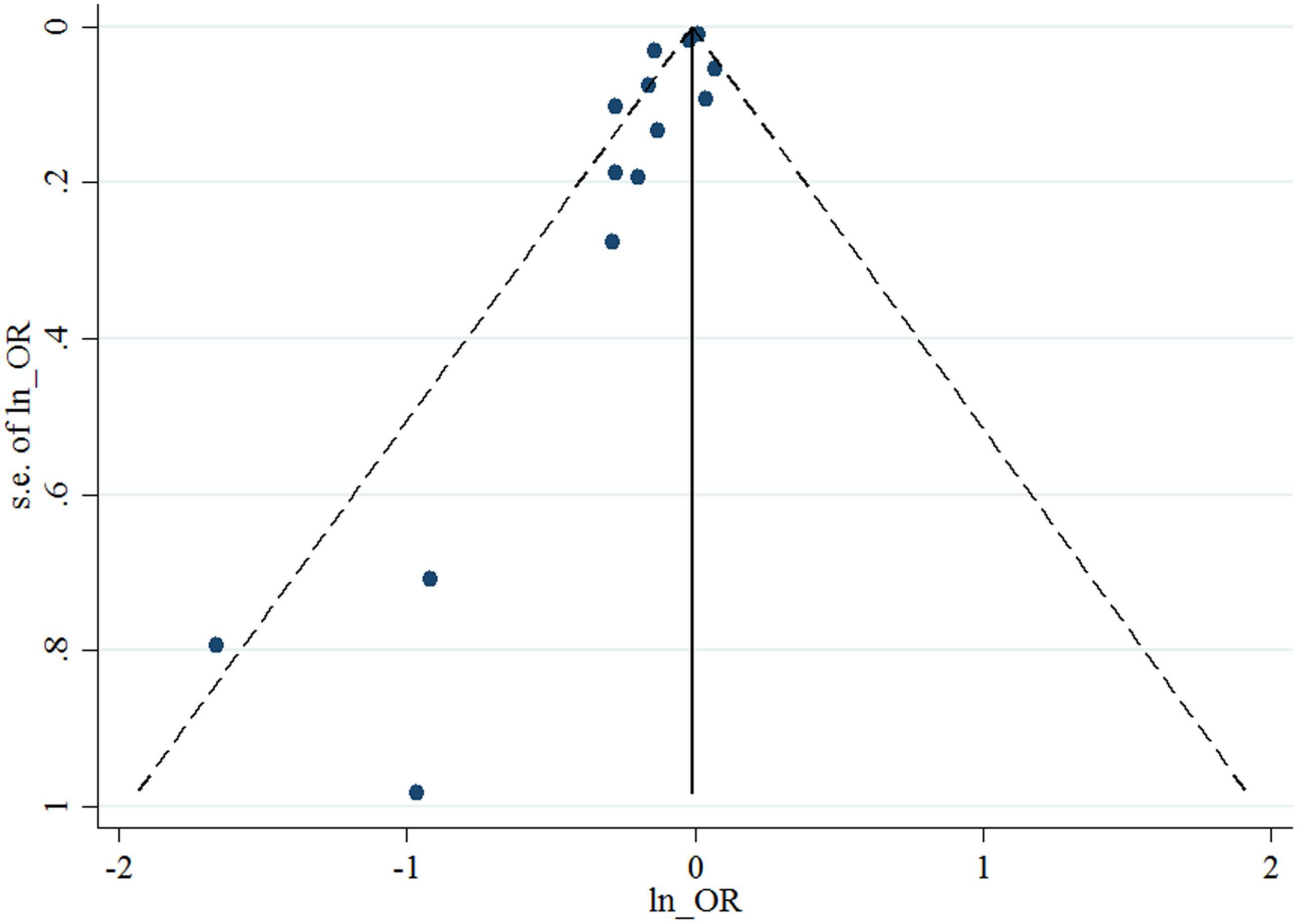
Funnel plot for the primary meta‐analysis of probiotic supplementation and pediatric UTI incidence or recurrence. Visual asymmetry was interpreted together with Egger's regression and Begg's rank‐correlation tests.

Visual inspection of the funnel plot suggested some asymmetry (Figure 4). However, neither Egger's regression test (*p* = 0.21) nor Begg's rank‐correlation test (*p* = 0.45) provided statistically significant evidence of small‐study effects. Funnel‐plot asymmetry can arise from mechanisms other than publication bias, including between‐study heterogeneity, clinical or methodological variability, and differences in study precision; conversely, formal asymmetry tests may have limited power with a relatively small number of studies. Thus, publication bias was not demonstrated but could not be confidently excluded.

### 3.7 Dose–Response Analysis of Probiotic Dose and UTI Incidence or Recurrence

**Figure 5:**
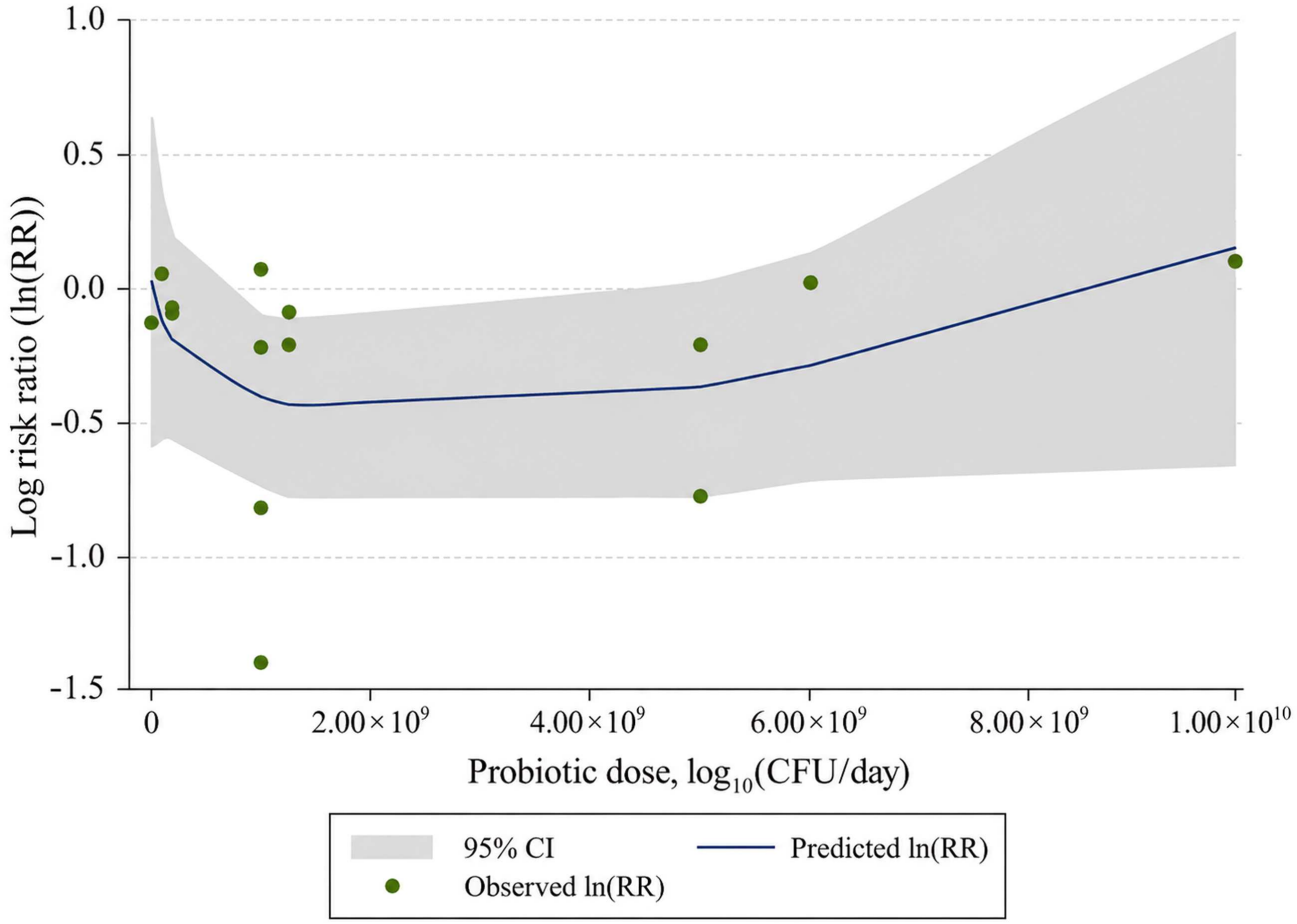
Exploratory dose–response relationship between total daily probiotic dose and the risk of pediatric UTI incidence or recurrence. Probiotic doses were harmonized as total daily viable counts (CFU/day) and modeled on a log10 scale. The solid line represents the predicted dose–response relationship and the shaded area represents the 95% confidence interval. CFU harmonization does not imply equivalent biological activity across probiotic strains or formulations.

The dose–response analysis included only studies in which total daily probiotic exposure could be reliably quantified in CFU/day. After harmonizing doses across dosing frequencies and formulations, no statistically significant nonlinear association was observed between total daily probiotic dose and the risk of UTI incidence or recurrence (P for nonlinearity = 0.15) (Figure 5). These findings should be interpreted cautiously because the probiotic preparations differed substantially in strain composition and formulation, and equivalent CFU counts do not necessarily represent equivalent biological activity across probiotic strains.

### 3.8 Grading of Evidence

The certainty of evidence for the primary outcome, UTI incidence or recurrence, was assessed using the GRADE framework. The evidence was downgraded by one level for risk of bias because several included trials had high or unclear risk in important methodological domains, including blinding, allocation procedures, incomplete outcome data, and selective reporting. A further one‐level downgrade was applied for inconsistency because substantial between‐study heterogeneity was observed (*I* ^2^ = 71.1%) and was not adequately explained by the available subgroup analyses. Indirectness was not considered serious because the included studies directly evaluated pediatric populations receiving probiotic supplementation for UTI incidence or recurrence. Imprecision was not downgraded because the pooled estimate was based on 3129 participants and the 95% CI was relatively narrow, although the clinical importance of the modest effect remains uncertain. Despite some visual funnel‐plot asymmetry, neither Egger's regression test (*p* = 0.21) nor Begg's rank‐correlation test (*p* = 0.45) provided statistically significant evidence of small‐study effects; therefore, publication bias was not considered sufficiently evident to warrant downgrading. Overall, the certainty of evidence was rated as low, primarily because of concerns regarding risk of bias and inconsistency (Table 3).

## 4 Discussion

This systematic review and meta‐analysis of 13 included studies involving 3129 pediatric participants found that probiotic supplementation was associated with a modest reduction in the overall risk of UTI incidence or recurrence (RR = 0.93, 95% CI: 0.88–0.99). However, substantial between‐study heterogeneity (*I* ^2^ = 71.1%) and low certainty of evidence warrant cautious interpretation of the pooled estimate. The clinical importance of the relative effect remains uncertain because no validated minimally important difference has been established for probiotic prevention of pediatric UTI, and clinical relevance depends on baseline risk and corresponding absolute effects. The findings are therefore best interpreted as an average association across heterogeneous clinical and intervention contexts rather than as a uniform treatment effect.

Exploratory subgroup analyses showed an RR of 0.95 (95% CI: 0.89–1.01) for UTI incidence and an RR of 0.89 (95% CI: 0.81–0.99) for UTI recurrence. However, the formal test for subgroup differences was not statistically significant (*p* = 0.28); consequently, these data do not establish that probiotic efficacy differs between UTI incidence and recurrence. Likewise, the exploratory dose–response analysis did not identify a statistically significant nonlinear association. These findings should not be used to infer preferential efficacy in a specific outcome category or an optimal probiotic dose, particularly given the marked heterogeneity in strains, formulations, clinical populations, comparators, and outcome definitions.

The substantial heterogeneity observed in the overall meta‐analysis (*I* ^2^ = 71.1%) likely reflects the combined influence of several clinical and intervention‐related sources of variability. The included trials enrolled markedly different pediatric populations, ranging from children after a first UTI to those with recurrent infection or vesicoureteral reflux, infants with acute pyelonephritis, critically ill children in pediatric intensive care units, and preterm infants. These populations differ in baseline UTI risk, underlying susceptibility, antibiotic exposure, and clinical setting. Considerable heterogeneity was also present across probiotic interventions, including differences in strain composition, single‐ versus multi‐strain formulations, viable microbial dose, dosing frequency, supplementation duration, and concomitant antimicrobial therapy. In addition, variation in UTI definitions, microbiological thresholds, and follow‐up periods may have contributed to differences in observed treatment effects.

Exploratory subgroup analyses provided some insight into this variability. Heterogeneity remained high among studies evaluating UTI incidence (*I* ^2^ = 80.5%) but was absent among studies evaluating UTI recurrence (*I* ^2^ = 0.0%). Nevertheless, the test for subgroup differences was not statistically significant, and subgrouping by supplementation duration also failed to materially explain the overall heterogeneity. Therefore, no single measured study characteristic could be identified as a definitive source of heterogeneity. Rather, the observed inconsistency most likely reflects the combined effects of differences in clinical populations, probiotic preparations, treatment protocols, outcome definitions, and study design. These findings support cautious interpretation of the pooled estimate as an average effect across heterogeneous clinical and intervention contexts rather than a uniform treatment effect applicable to all pediatric populations or probiotic formulations.

Additional sources of heterogeneity were related to the comparator groups, UTI definitions, and duration of follow‐up. Comparator conditions varied considerably across trials and included placebo or no probiotic supplementation, standard care, and different antibiotic regimens, including nitrofurantoin and trimethoprim–sulfamethoxazole. Such differences are clinically relevant because the baseline preventive effect of the comparator may modify the apparent incremental benefit attributable to probiotic supplementation. In addition, the diagnostic criteria used to define UTI were not uniform across studies. Some trials relied primarily on microbiological thresholds, with significant bacteriuria defined using cutoffs ranging from approximately 10^3^ to 10^5^ CFU/mL depending on the urine collection method, whereas others incorporated clinical symptoms, study‐specific definitions, or combinations of clinical and microbiological criteria. Variation in outcome ascertainment may therefore have contributed to differences in event classification and observed treatment effects. Follow‐up duration also varied substantially, ranging from very short inpatient periods to several months or up to 2 years, which may have influenced the opportunity to detect incident or recurrent UTIs. Taken together, variability in comparator intensity, diagnostic thresholds, outcome definitions, and follow‐up duration likely contributed to the substantial between‐study heterogeneity observed in the overall analysis and further supports cautious interpretation of the pooled estimate as an average effect across clinically diverse settings.

The modest reduction in UTI risk observed in the present meta‐analysis is biologically plausible, although the clinical findings alone cannot establish the underlying mechanisms. Probiotic effects are increasingly recognized as strain‐specific and may extend beyond direct competition with potential pathogens. Proposed mechanisms include modulation of microbial community structure, production of bioactive microbial metabolites, reinforcement of epithelial barrier integrity, and regulation of mucosal and systemic immune responses [6]. Adhikary et al. recently summarized evidence indicating that probiotic‐ and synbiotic‐derived metabolites can influence inflammatory pathways, redox homeostasis, epithelial barrier function, and host immune regulation [7]. Such mechanisms may theoretically enhance resistance to pathogen colonization and support mucosal host defense [30, 31]. In the context of UTI, additional proposed pathways include interference with uropathogen adhesion and competition for ecological niches within interconnected intestinal and urogenital microbial ecosystems. Importantly, however, these mechanistic observations are derived largely from broader experimental and translational literature and should not be interpreted as direct evidence that probiotics prevent pediatric UTIs [32, 33].

Compared with earlier reviews of probiotics for pediatric UTI prevention, the present synthesis adds an exploratory dose–response analysis and a formal GRADE assessment of evidence certainty. Meena et al. (2021) [9] and Hosseini et al. (2017) [5] likewise reported heterogeneous findings across probiotic interventions, underscoring persistent uncertainty about strain‐specific efficacy and the influence of concomitant antibiotic therapy. The additional analyses in the present review improve characterization of the evidence base but do not overcome the substantial clinical heterogeneity or the limitations of the underlying studies; accordingly, they should not be interpreted as establishing an optimal probiotic regimen or a definitive class effect.

From a public‐health perspective, interest in probiotic and other non‐antibiotic preventive strategies is particularly relevant in the context of increasing antimicrobial resistance and the cumulative antibiotic exposure associated with recurrent pediatric UTIs. A safe intervention capable of reducing even a proportion of recurrent infections could potentially contribute to antibiotic‐sparing approaches in appropriately selected patients. Nevertheless, the present findings do not support replacing established antibiotic treatment or indicated prophylaxis with probiotics. Rather, the modest pooled effect, substantial between‐study heterogeneity, absence of a clear dose–response relationship, and variability in probiotic strains and formulations suggest that probiotics should currently be considered an investigational or adjunctive strategy rather than a standardized alternative to antibiotic prophylaxis [34].

This review has several strengths, including adherence to PRISMA 2020 reporting standards, inclusion of randomized controlled trials from diverse geographic and clinical settings, formal assessment of the certainty of evidence using GRADE, and an exploratory dose–response analysis intended to examine whether probiotic exposure was associated with variation in UTI risk. These features provide a structured and clinically informative synthesis of the available evidence.

Nevertheless, several limitations should be considered when interpreting the findings. Most importantly, the included trials exhibited substantial clinical and biological heterogeneity in probiotic strain composition, single‐ versus multi‐strain formulations, viable microbial dose, dosing frequency, route and duration of administration, underlying UTI risk, concomitant antibiotic use, and follow‐up. Accordingly, the pooled estimate should be interpreted as an average class‐level effect across heterogeneous probiotic interventions rather than as evidence that individual strains or formulations have equivalent efficacy. This distinction is particularly important because probiotic effects are likely to be strain‐specific and may depend on formulation, viability, dose, host characteristics, and treatment context. The same limitation applies to the dose–response analysis. Although harmonization to total daily CFU permitted quantitative comparison of microbial exposure across studies, equivalent CFU counts do not necessarily indicate equivalent biological potency across different strains or products. Moreover, some studies used weight‐based or incompletely reported dosing regimens for which a fixed daily CFU exposure could not be derived reliably and were therefore not included in the dose–response model. Consequently, the dose–response findings should be considered exploratory and hypothesis‐generating rather than evidence of an optimal dose applicable across probiotic products. Additional limitations include methodological concerns related to blinding and outcome reporting in some trials, variation in follow‐up duration, and inconsistency in UTI definitions, with several studies relying predominantly on microbiological criteria rather than standardized combinations of clinical symptoms and culture‐confirmed infection. These differences may have contributed to between‐study heterogeneity and limited the direct comparability of outcomes. The substantial statistical heterogeneity (*I* ^2^ = 71.1%) further limits the precision with which the overall pooled effect can be generalized. Although exploratory subgroup analyses suggested that heterogeneity was concentrated primarily among studies evaluating UTI incidence, neither UTI outcome type nor supplementation duration statistically accounted for the overall between‐study variability, suggesting that multiple clinical, intervention‐related, and methodological factors contributed simultaneously.

Finally, the review was not prospectively registered and no separate protocol was published, limiting independent assessment of deviations from the originally planned methods. Taken together, these considerations warrant cautious interpretation of the modest pooled effect and highlight the need for adequately powered, prospectively registered trials evaluating well‐characterized, strain‐specific probiotic preparations using standardized dosing, outcome definitions, and follow‐up procedures.

Future trials should move beyond treating probiotics as a homogeneous intervention. Adequately powered RCTs should evaluate well‐characterized, strain‐specific preparations using standardized CFU reporting, clinically relevant dosing schedules, harmonized UTI definitions, and sufficiently long follow‐up. Incorporating microbiome profiling, microbial metabolite measurements, and markers of mucosal or systemic immune function may also help determine whether observed clinical effects are mediated through the biological pathways proposed in contemporary mechanistic literature. Such studies would be particularly valuable in children with recurrent UTI, in whom the potential benefit of an effective non‐antibiotic adjunct may be greatest.

## 5 Conclusion

The available evidence suggests that probiotic supplementation may be associated with a small reduction in the risk of UTI incidence or recurrence in pediatric populations. However, substantial clinical and statistical heterogeneity, variation in probiotic preparations and comparator regimens, inconsistent UTI definitions and follow‐up periods, and low certainty of evidence limit the strength and generalizability of this finding. Exploratory subgroup and dose–response analyses do not establish differential efficacy by UTI outcome type or an optimal probiotic dose. Further well‐designed, prospectively registered studies using clearly characterized strain‐specific interventions and standardized outcome definitions are needed before firm clinical recommendations can be made.

## Author Contributions

**Sayed Yousef Mojtahedi:** conceptualization, investigation, methodology. **Masoumeh Ghasempour:** investigation, methodology, formal analysis. **Maryam Ghodsi:** formal analysis, data curation. **Paniz Pourpashang:** conceptualization, investigation, writing – original draft, writing – review and editing.

## Funding

The authors have nothing to report.

## Ethics Statement

Not applicable. This systematic review and meta‐analysis used published aggregate data and involved no new recruitment or collection of identifiable participant‐level information; therefore, institutional ethics approval and informed consent were not required.

## Consent

The authors have nothing to report.

## Conflicts of Interest

The authors declare no conflicts of interest.

## Clinical Trial Registration

NA

## Transparency Statement

Paniz Pourpashang, the corresponding author and manuscript guarantor, affirms that this manuscript is an honest, accurate, and transparent account of the study being reported; that no important aspects of the study have been omitted; and that any discrepancies from the study as planned (and, if relevant, registered) have been explained.

## Supporting information


Supporting File

## Data Availability

The original contributions presented in the study are included in the article, further inquiries can be directed to the corresponding author/s. The authors confirm that the data supporting the findings of this study are available within the article and its supporting materials. No new individual‐participant data were generated by this systematic review and meta‐analysis.
