# Supplementary material for: Efficacy of Probiotic Supplementation for Preventing Pediatric Urinary Tract Infections: A GRADE‐Based Dose–Response Meta‐Analysis
Source: Health Sci Rep. 2026 Sep 1;9(9):e73107. doi: 10.1002/hsr2.73107 (PMC13534828; doi:10.1002/hsr2.73107)
Supplement: Supplementary file 1 [file HSR2-9-e73107-s001.docx]

**Supplementary Material**

Supplementary Table S1. Full electronic search strategies used to identify eligible studies.

**Search coverage:** January 1, 1990 through June 5, 2025. **Language restrictions:** none.

| **Database** | **Search strategy** | **Records retrieved** |
| --- | --- | --- |
| **PubMed** | ((((("Urinary Tract Infections"[MeSH]) OR ("Cystitis"[MeSH]) OR ("Pyelonephritis"[MeSH])) OR (urinary tract infect* [tiab] OR UTI[tiab] OR UTIs[tiab] OR cystitis[tiab] OR pyelonephritis[tiab]))) AND (((("Probiotics"[MeSH]) OR ("Lactobacillus"[MeSH]) OR ("Bifidobacterium"[MeSH]) OR ("Saccharomyces"[MeSH])) OR (probiotic*[tiab] OR synbiotic*[tiab] OR symbiotic*[tiab] OR lactobacillus[tiab] OR bifidobacterium[tiab] OR saccharomyces[tiab]))) AND (((("Child"[MeSH]) OR ("Infant"[MeSH]) OR ("Adolescent"[MeSH])) OR (child*[tiab] OR infan*[tiab] OR newborn*[tiab] OR baby[tiab] OR babies[tiab] OR pediatric*[tiab] OR paediatric*[tiab] OR adolescen*[tiab] OR teen*[tiab] OR boy*[tiab] OR girl*[tiab])))) | 173 |
| **Scopus** | ( TITLE-ABS-KEY ( "urinary tract infect*" OR uti OR utis OR cystitis OR pyelonephritis ) ) AND ( TITLE-ABS-KEY ( probiotic* OR synbiotic* OR symbiotic* OR lactobacillus OR bifidobacterium OR saccharomyces ) ) AND ( TITLE-ABS-KEY ( child* OR infan* OR newborn* OR baby OR babies OR pediatric* OR paediatric* OR adolescen* OR teen* OR boy* OR girl* ) ) | 490 |
| **Web of Science** | TS=("urinary tract infect*" OR UTI OR UTIs OR cystitis OR pyelonephritis) AND TS=(probiotic* OR synbiotic* OR symbiotic* OR lactobacillus OR bifidobacterium OR saccharomyces) AND TS=(child* OR infan* OR newborn* OR baby OR babies OR pediatric* OR paediatric* OR adolescen* OR teen* OR boy* OR girl*) | 160 |
| **EMBASE** | 'probiotic'/exp OR probiotic*:ti,ab,kw OR synbiotic*:ti,ab,kw OR symbiotic*:ti,ab,kw OR lactobacillus:ti,ab,kw OR bifidobacterium:ti,ab,kw OR saccharomyces:ti,ab,kw AND 'urinary tract infection'/exp OR 'cystitis'/exp OR 'pyelonephritis'/exp OR 'urinary tract infect*':ti,ab,kw OR uti:ti,ab,kw OR utis:ti,ab,kw OR cystitis:ti,ab,kw OR pyelonephritis:ti,ab,kw AND 'child'/exp OR 'infant'/exp OR 'newborn'/exp OR 'adolescent'/exp OR child*:ti,ab,kw OR infan*:ti,ab,kw OR newborn*:ti,ab,kw OR baby:ti,ab,kw OR babies:ti,ab,kw OR pediatric*:ti,ab,kw OR paediatric*:ti,ab,kw OR adolescen*:ti,ab,kw OR teen*:ti,ab,kw OR boy*:ti,ab,kw OR girl*:ti,ab,kw | 325 |

**Note.** Synbiotic* and symbiotic* were intentionally included as sensitivity-oriented retrieval terms to maximize search sensitivity and identify records that might contain a separable probiotic-only intervention or probiotic-specific data. Their inclusion in the search strategy did not broaden the eligibility criteria; synbiotic-only interventions were excluded unless the probiotic effect could be isolated.

**Abbreviations:** MeSH, Medical Subject Headings; UTI, urinary tract infection.

Supplementary Figure S1. Exploratory subgroup analysis according to intervention duration.

**Note.** The two intervention-duration categories and study assignments are reproduced from the supplied statistical output. Pooled estimates are shown with 95% confidence intervals under a random-effects model.

Supplementary Figure S2. Leave-one-out sensitivity analysis of the overall pooled effect.

**Note.** Each row represents the pooled estimate after sequential omission of the named study. The original statistical graphic was retained to avoid altering underlying numerical information.
